# Aberrant Regulation of HDAC2 Mediates Proliferation of Hepatocellular Carcinoma Cells by Deregulating Expression of G1/S Cell Cycle Proteins

**DOI:** 10.1371/journal.pone.0028103

**Published:** 2011-11-23

**Authors:** Ji Heon Noh, Kwang Hwa Jung, Jeong Kyu Kim, Jung Woo Eun, Hyun Jin Bae, Hong Jian Xie, Young Gyoon Chang, Min Gyu Kim, Won Sang Park, Jung Young Lee, Suk Woo Nam

**Affiliations:** Department of Pathology, College of Medicine and Functional RNomics Research Center, The Catholic University of Korea, Seoul, Korea; Centro Cardiologico Monzino, Italy

## Abstract

Histone deacetylase 2 (HDAC2) is crucial for embryonic development, affects cytokine signaling relevant for immune responses and is often significantly overexpressed in solid tumors; but little is known about its role in human hepatocellular carcinoma (HCC). In this study, we showed that targeted-disruption of HDAC2 resulted in reduction of both tumor cell growth and *de novo* DNA synthesis in Hep3B cells. We then demonstrated that HDAC2 regulated cell cycle and that disruption of HDAC2 caused G1/S arrest in cell cycle. In G1/S transition, targeted-disruption of HDAC2 selectively induced the expression of p16^INK4A^ and p21^WAF1/Cip1^, and simultaneously suppressed the expression of cyclin D1, CDK4 and CDK2. Consequently, HDAC2 inhibition led to the down-regulation of E2F/DP1 target genes through a reduction in phosphorylation status of pRb protein. In addition, sustained suppression of HDAC2 attenuated *in vitro* colony formation and *in vivo* tumor growth in a mouse xenograft model. Further, we found that HDAC2 suppresses p21^WAF1/Cip1^ transcriptional activity via Sp1-binding site enriched proximal region of p21^WAF1/Cip1^ promoter. In conclusion, we suggest that the aberrant regulation of HDAC2 may play a pivotal role in the development of HCC through its regulation of cell cycle components at the transcription level providing HDAC2 as a relevant target in liver cancer therapy.

## Introduction

Acetylation of histones and non-histone proteins pivotally modulates gene expression and cell signaling. Histone deacetylases (HDACs), which remove acetyl groups from hyper-acetylated histones, counteract the effects of histone acetyltransferases (HATs) and return histone to its basal state with the concomitant suppression of gene transcription. There are 18 HDACs which are generally divided into four classes based on sequence homology to yeast counterparts [1], [2]. There is clear evidence for the involvement of both HATs and HDACs in cell proliferation, differentiation and cell cycle regulation [3], [4]. Moreover, it has been reported that the pathological activity and deregulation of HDACs can lead to several diseases such as cancer, immunological disturbances and muscular dystrophies [2], [5]. A multitude of HDAC inhibitors have been developed and are currently tested as anticancer agents in various solid and hematologic malignancies. However, the HDAC inhibitors used to demonstrate effects on cells are for the most part non-specific for the different HDAC isoforms [6]. In addition, although there are clear evidences for the involvement of HDACs in the development of cancer, the specific roles of individual HDAC in the regulation of cell proliferation and apoptosis are unclear.

A previous study has suggested that HDAC2 expression was increased by the loss of APC in human colorectal cancer [7]. We have also reported that overexpression of HDAC2 was found in stomach and liver cancer [8], [9]. Moreover, we have noted that HDAC2 expression was gradually increased from non-tumor to overt cancer based on gene expression analysis of multi-step histopathological grades of HCC (Figure S1) [10]. These results suggest that HDAC2 plays an important role in the development and progression of those cancers. In fact, there were some reports suggesting that inhibition of HDACs using various known HDAC inhibitors exhibited antitumor activities and/or apoptotic effects in HCC model systems including HCC-derived cell lines or murine models [11], [12]. Furthermore, a recent report suggested that HDAC inhibitor enhanced the sensitivity towards cell death-mediated apoptosis such as TNF-related apoptosis inducing ligand [13]. However, the underlying molecular mechanisms of HDAC inhibition in HCC remain largely unknown. Also, no attempts have been made so far to explain the cellular mechanisms responsible for the mitogenic potential of HDAC2 in HCC.

In the present study, to investigate biological roles of HDAC2 that confer oncogenic potential in human HCC, we assessed the aberrant regulation of HDAC2 in human HCC and examined the regulatory mechanisms of HDAC2 in cell cycle of HCC cells. In addition, *in vitro* and *in vivo* experimental tumorigenic potential of HDAC2 were explored using stable HDAC2 knockdown cell lines.

## Results

### Aberrant regulation of HDAC2 is independent of Wnt pathway and c-Myc in HCC

A previous study demonstrated that the increased HDAC2 expression was found in colon cancer, and the induction of HDAC2 was dependent on Wnt pathway and c-Myc [7]. Our previous report also showed the overexpression of HDAC2 in human HCCs [8], [10]. It has been reported that activation of Wnt pathway in hepatocarcinogenesis can be caused by a stabilizing mutation of *β-catenin* gene (15∼25% of cases) or by an inactivating mutation of *Axin1* gene (5% of cases) [14]. This fact raises a possibility that increased HDAC2 expression could also be regulated by Wnt pathway in HCC progression.

**Figure 1:**
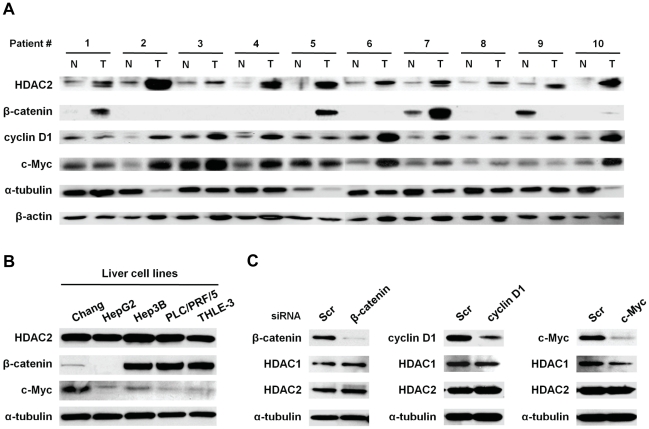
Overexpression of HDAC2 is not regulated by Wnt signaling in human HCC. (A) Tissue lysates were prepared from non-tumoral liver tissues (N) or tumors (HCC, HBV-positive, Edmondson grade G3) (T) and immunoblotted with indicated antibodies. (B) Protein expressions of HDAC2, β-catenin and c-Myc were also determined by immunoblotting in human liver cancer cell lines. (C) Hep3B cells were transfected with siRNAs targeting β-catenin, cyclin D1 or c-Myc. The protein level of HDAC1 or 2 was assessed by immunoblotting. A typical result from three performed experiments is shown.

Thus, to identify the aberrant expression of HDAC2 and correlate its regulation with Wnt pathway, we performed immunoblotting of HDAC2, β-catenin, cyclin D1 and c-Myc in a subset of 10 paired human HCCs. As shown in Figure 1A, HDAC2 appeared to be highly overexpressed in all 10 HCC tissues compared to the corresponding non-cancerous tissues. However, up-regulation of β-catenin was observed in 3 cases (patient# 1, 5 and 7) out of the 10 examined HCCs. This frequency of β-catenin overexpression (30%) is consistent with previous reports on the activation of Wnt signaling by stabilization of β-catenin in HCC [14]. In contrast, cyclin D1 and c-Myc appeared to be overexpressed in almost all HCCs compared to non-tumor controls.

We next investigated protein expressions related to Wnt signaling in human liver cancer cell lines including THLE-3, a normal immortalized hepatocyte cell line. High level of HDAC2 expression was detected in all tested cell lines, while β-catenin was not detectable in HepG2, a hepatoblastoma cell line or c-Myc expression varied in different liver cell lines (Figure 1B). These suggest that activation of Wnt signaling and/or c-Myc may not contribute to aberrant regulation of HDAC2 in liver cancers. Finally, this suggestion was further validated by gene knockdown of *β-catenin*, *cyclin D1* and *c-Myc* in Hep3B cells. As expected, HDAC2 expression was not affected by depletion of β-catenin, cyclin D1 or c-Myc in Hep3B cells (Figure 1C). These results indicated that aberrant regulation of HDAC2 is not dependent on Wnt pathway or c-Myc activation in liver tumorigenesis.

### Targeted-disruption of HDAC2 elicits anti-mitogenic effects on HCC

**Figure 2:**
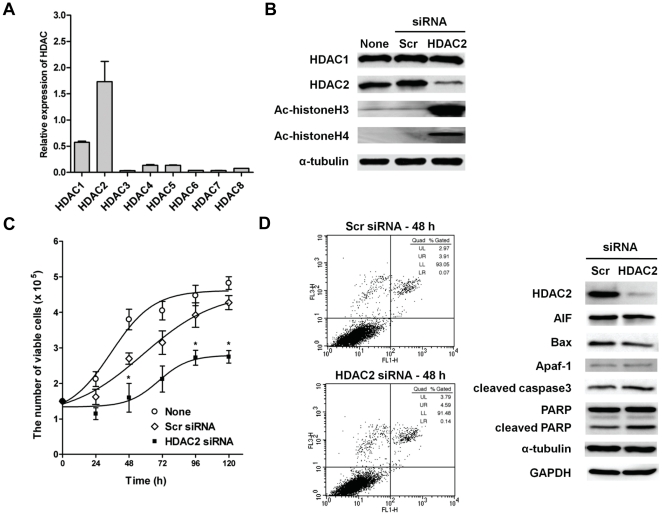
Effects of HDAC2 inhibition on the cell growth and apoptosis. (A) Endogenous expression of HDACs (HDAC1-8) in Hep3B cells by qRT-PCR. Each HDAC mRNA level was normalized against GAPDH. (B) HDAC2 was depleted by HDAC2 specific siRNA in Hep3B cells. To ascertain the knockdown efficiency and suppression of HDAC activity, protein expressions of HDAC2, acetyl-histone H3 and H4 were determined by immunoblotting. (C) Time course analysis of the effect of HDAC2 inhibition on adherent cell number. The cell counts show the results of four independent experiments, expressed as mean ± SD (* p<0.05 relative to Scr siRNA). (D) After 48 h transfection of HDAC2 siRNA, cells were stained with Annexin V-FITC and propidium iodide, and apoptotic cells were analyzed by flow cytometric analysis (left). Protein expressions of the proapoptotic genes (AIF, Bax, Apaf-1, cleaved-caspase3, cleaved-PARP) were also determined by immunoblotting (right).

We next investigated that HDAC2 represented major histone deacetylase activity among the HDACs in HCC. We assessed the endogenous expression levels of various HDACs in Hep3B cells by using qRT-PCR with specific primer for each HDAC (Table S3). HDAC2 resulted in the highest expression level among the analyzed HDACs (Figure 2A). In addition, HDAC2 depletion caused accumulation of acetylated histones H3 and H4 indicating a major deacetylase activity in Hep3B cells (Figure 2B).

Next, to explain the biological consequences of aberrant expression of HDAC2 in hepatocarcinogenesis, HDAC2 expression was abrogated by small interfering RNA (siRNA) directed against HDAC2. The growth rate of cells transfected with HDAC2 siRNA was significantly regressed compared to non- (None) or scrambled siRNA transfectants (Scr) (Figure 2C). To generalize this finding to liver cancer cells, we employed three more different liver cancer cell lines and investigated the tumor suppressing effects of HDAC2-targeting upon tumor cell growth. Among liver cancer cell lines, we selected HepG2, SNU-182 and SNU-449 cell lines as they exhibited relatively high expression for HDAC2 in Western blot analyses (Figure S2A). Then, each cell line was transfected with HDAC2 siRNA. As shown in Figure S2B, the growth rate of HDAC2 knockdown cells appeared to be significantly regressed as compared to control (None or control siRNA transfectant) cells. Concordant with previous observation in Hep3B cells, all cell lines displayed significant growth retardation upon HDAC2-targeting. These results strengthen our suggestion that aberrant overexpression of HDAC2 contributes mitotic potential during liver tumorigenesis. The anti-growth effect of HDAC2 depletion in HCC cells could be partially explained by the intervention of cell growth regulation such as cellular apoptosis, cell cycle arrest or cellular senescence. Thus, we next explored the effects of HDAC2 suppression on cellular apoptosis and cell cycle regulation. Flow cytometric analysis for measuring Annexin V stained cells showed no significant induction of apoptotic cells compared to control (Scr siRNA) (Figure 2D, left panel). In addition, HDAC2 depletion did not affect expressions of pro-apoptotic components such as AIF, Bax and Apaf-1, nor did it cause caspase-3 or PARP cleavage (Figure 2D, right panel). These results indicated that overexpression of HDAC2 did not affect the apoptotic signal in liver tumorigenesis.

**Figure 3:**
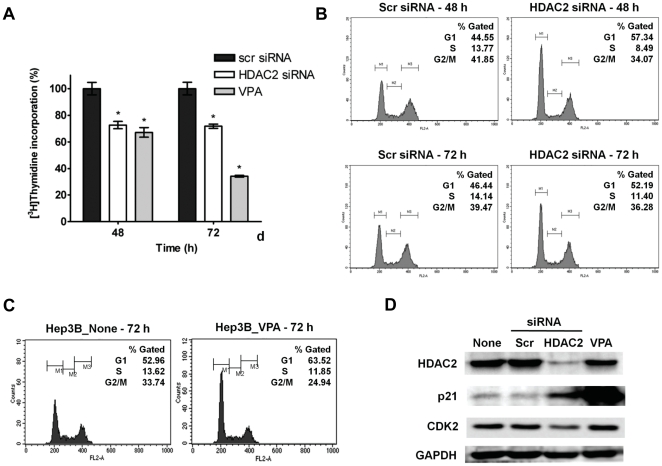
Effects of HDAC2 inhibition on the proliferation and the cell cycle progression of HCC cells. (A) Cell proliferating activity was assessed by thymidine incorporation analysis in Hep3B cells after transfection with HDAC2 or scrambled siRNA. At 48 and 72 post-transfection, [^3^H]-thymidine incorporation was analyzed by scintillation counting. The results represent the mean ± SD of three experiments (* p<0.01). (B) After 48 and 72 h post-transfection of HDAC2 siRNA, the DNA content of PI-stained cells was analyzed by flow cytometry. (C) After 1 mM Valproic acid treatment to Hep3B cells for 72 h, the DNA content of PI-stained cells was analyzed by flow cytometry. (D) Hep3B cells were transient transfected with HDAC2 or scrambled siRNA (100 nM) for 72 h, and 1 mM of Valproic acid was also treated on Hep3B cells for 72 h. The protein expressions of p21^WAF1/Cip1^ and CDK2 were determined by immunoblotting. Three experiments with the same results were performed.

We then investigated if HDAC2 depletion causes anti-mitogenic effect in HCC cells. As shown in Figure 3A, *de novo* DNA synthesis was significantly inhibited by HDAC2 depletion at both 48 and 72 h post-transfection of HDAC2 siRNA compared to control in thymidine incorporation assay. This anti-proliferative effect of HDAC2 suppression was compared to the treatment with a potent HDAC inhibitor, VPA on Hep3B cells (Figure 3A). We then performed cell cycle analysis of PI-stained cells in HDAC2 siRNA transfectants using flow cytometry. HDAC2 depletion led to an increase in G1 phase by 12.8% with a concomitant decrease in S phase and G2/M phase by 5.3% and 7.8% respectively at 48 h after transfection (Figure 3B). VPA also appeared to induce cell cycle arrest in G1 phase with strong activation of p21^WAF1/Cip1^ expression in Hep3B cells (Figs. 3C and 3D). Similarly, HDAC2 depletion also caused induction of p21^WAF1/Cip1^ expression in Hep3B cells. However, interestingly, we found that HDAC2 depletion also suppressed cyclin-dependent kinase 2 (CDK2) expression, but VPA did not in Hep3B cells (Figure 3D).

### Selective regulation of HDAC2 on G1/S components of cell cycle circuitry in Hep3B cells

The fact that the suppression of HDAC2 caused cell cycle arrest in G1 phase implies that HDAC2 can modulate activities of cell cycle regulating components. Therefore, we examined the effects of HDAC2 depletion on the regulatory components of G1/S cell cycle transition. In G1/S transition, it has been well established that negative cell cycle regulators such as p15^INK4B^, p16^INK4A^, p18^INK4C^, p19^INK4D^, p27^Kip1^ and p21^WAF1/Cip1^ are the key modulators that suppress cyclin D1/CDK4, 6 or cyclin E/CDK2 complexes [15].

**Figure 4:**
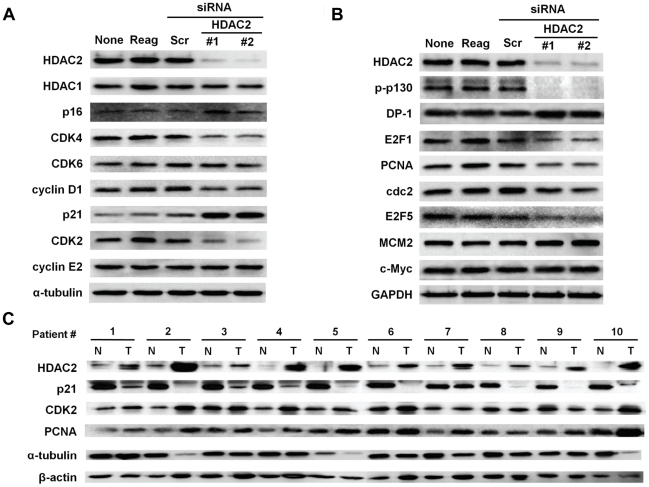
Systemic modulation of regulatory components by HDAC2 in G1/S cell cycle transition. Hep3B cells were transfected with no siRNA (None), oligofectamine only (Reag), 100 nmol/L scrambled siRNA (Scr) or 100 nmol/L of two different HDAC2 specific siRNAs (#1 and #2), and were harvested at 72 h post-transfection. (A) Immunoblotting of CDKs, cyclins and CDK inhibitors of G1/S transition were performed. (B) Effects of HDAC2 depletion on pRb and E2F/DP1 target gene expressions. (C) Protein expressions of p21^WAF1/Cip1^, CDK2 and PCNA in human HCC tissues were analyzed by immunoblotting. All experiments were repeated three times with same results.

When these cell cycle modulators were examined, p16^INK4A^ and p21^WAF1/Cip1^ were selectively induced by HDAC2 depletion in Hep3B cells. In addition, HDAC2 depletion also elicited concomitant suppression of cyclin D1, CDK4 and CDK2, respectively (Figure 4A). These results strongly suggest that HDAC2 overexpression causes the suppression of the negative cell cycle modulators such as p16^INK4A^ and p21^WAF1/Cip1^, and at the same time, induces the expression of their specific regulators such as cyclin D1, CDK4 and CDK2 in the G1/S transition of Hep3B cells.

In general, activated cyclin/CDK complex can cause hyperphosphorylation of pRb which loses its tumor suppressor activity, and which allows for E2F/DP1 transcriptional activity. Thus, we next investigated whether the dysregulation of cyclins and CDKs by HDAC2 affects the E2F/DP1 transcriptional activity. As expected, HDAC2 suppression caused hypophosphorylation of p130, a pRb isoform (Rb2) in Hep3B cells (Figure 4B). This result implies that the aberrant regulation of HDAC2 affects phosphorylation of Rb protein via transcriptional activation of cyclin/CDKs and/or inactivation of negative modulators. This suggestion was supported by the fact that the E2F/DP1 target genes such as E2F1, PCNA, CDC2 and E2F5 were selectively down-regulated by HDAC2 depletion (Figure 4B). This systemic regulation on cell cycle components was validated in 10 paired HCC tissues. As a result, p21^WAF1/Cip1^ expression was detected at relatively high levels in all non-tumor tissues, but it was greatly reduced or non-detectable in all HCC tissues. In contrast, CDK2 and PCNA appeared to be up-regulated in all HCC tissues compared to non-tumor tissues (Figure 4C).

**Figure 5:**
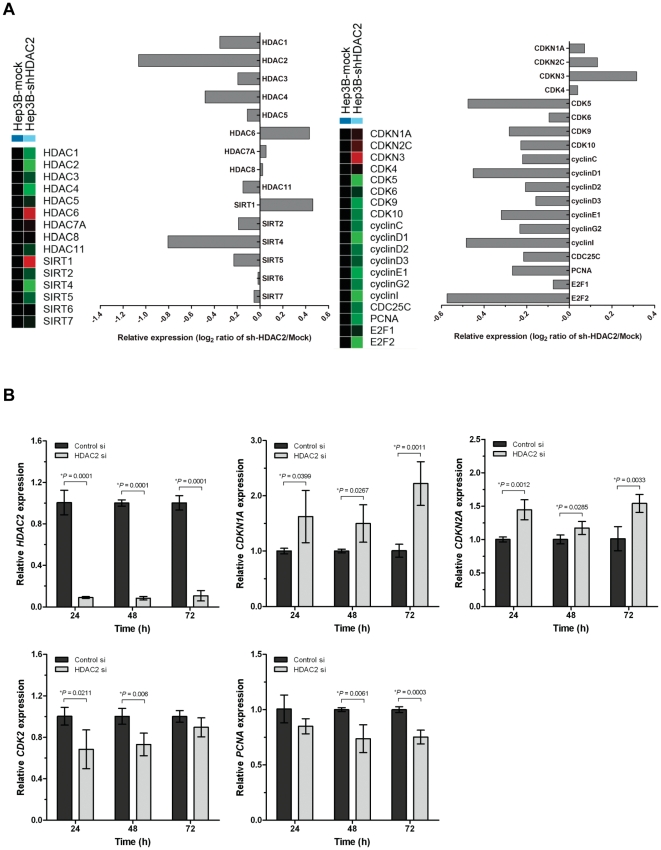
Identification of large-scale gene expression changes by HDAC2 inactivation in Hep3B cells. (A) Heatmap analysis of genes recapitulated for HDAC family and cell cycle regulation, and graphical representation of the expression changes of genes functionally involved in HDAC gene families (left) and cell cycle regulation (right) by HDAC2 inactivation of Hep3B cells. (B) Validation of microarray data by quantitative real-time PCR. The relative expression level of each gene was normalized to GAPDH mRNA in the same sample. The result of four independent experiments was shown as mean ± SD (Student's t-test).

These results indicate that HDAC2 inactivation may cause comprehensive gene expression changes of genetic elements that associate with mitotic activity of tumor cells. Thus, to identify the molecular signature that may affect tumor cell growth, we employed and performed large-scale gene expression analysis on HDAC2 knockdown cells (Hep3B-shHDAC2) and compared with its corresponding control (Hep3B-mock) by using whole genome expression microarray. From this, we could identify large number genetic elements that are differentially expressed by HDAC2 inactivation in Hep3B cells. Among these, we recapitulated genes that are associated with cell cycle regulation, and depicted their differential gene expressions as heat map and bar graphs (Figure 5A). As shown in Figure 5A, we could confirm HDAC2 inactivation by detecting very low endogenous HDAC2 expression level among HDACs and sirtuins (left panel). We also observed up-regulation of some negative cell cycle regulators such as *CDKN1A* (p21^WAF1/Cip1^), *CDKN2*C (p18^INK4C^), *CDKN3* genes, and simultaneously, down-regulation of some CDKs and cyclin genes and E2F/DP1 target genes such as *CDC25C, PCNA* and *E2F1* (Figure 5A, right panel). These results are consistent with the results in Figure 4 that show systemic modulation of cell cycle proteins by HDAC2 via Western blot analysis in Hep3B cells. Further, to validate gene expression data of microarrays and to confirm transcriptional levels of differentially expressed genes, we performed quantitative real-time PCR (qRT-PCR) for *HDAC2, CDKN1A* (p21^WAF1/Cip1^), *CDKN2A* (p16^INK4A^), *CDK2* and *PCNA* genes (Figure 5B). As shown in Figure 5B, Hep3B-siHDAC2 cells exhibited very low endogenous HDAC2 expression. With this condition, we were able to confirm that both CDKN1A (p21^WAF1/Cip1^) and CDKN2A (p16^INK4A^) were significantly up-regulated in time dependent manner. Inversely, CDK2 and PCNA were appeared to be down-regulated by HDAC2 inactivation. These results support our suggestion that cooperative suppression of p16^INK4A^ and p21^WAF1/Cip1^ and induction of CDK2, CDK4 and cyclin D1 expression by HDAC2 may exert a very potent mitotic stimulation causing uncontrolled cell growth during HCC progression.

### Sustained suppression of HDAC2 attenuates tumorigenic potential of Hep3B HCC cells in vitro and in vivo

**Figure 6:**
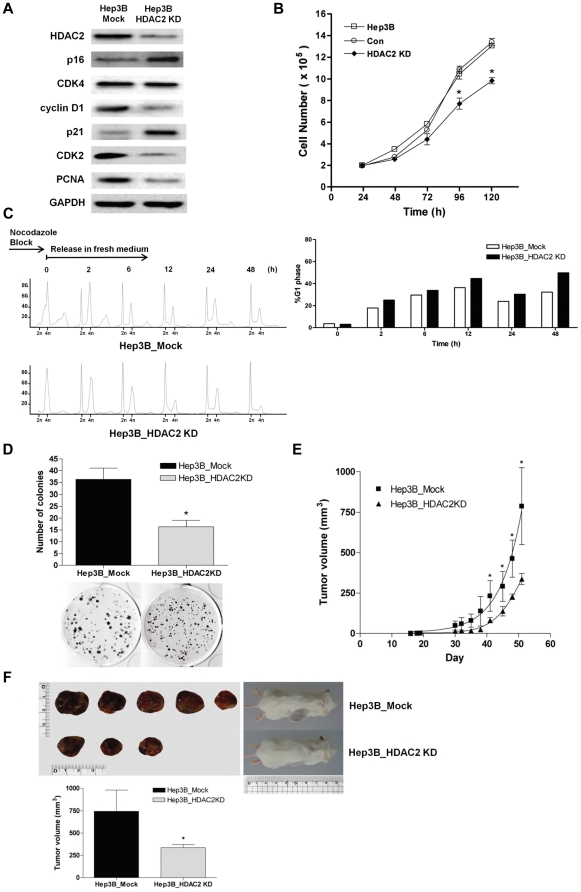
Sustained suppression of HDAC2 attenuates the tumorigenic potential of Hep3B cells *in vitro* and *in vivo*. (A) Confirmation of HDAC2 suppression by its specific regulation of cell cycle components in HDAC2 depleted cell lines. A typical result of three performed experiments is shown. (B) Time course cell counting analyses of Hep3B and two stable cell lines (Hep3B_Mock and Hep3B_HDAC2KD). The cell counts show the results of four independent experiments. (C) HDAC2-deficient (Hep3B_HDAC2KD) or Mock (Hep3B_Mock) stable cell line was blocked in G2/M transition by nocodazole and then released in fresh medium. The DNA content was determined by FACS analysis with PI-stained cells at each time point (0, 2, 6, 12, 24, 48 h after release) (left). The percentage of cells in G1 phase was calculated and represented as bar graph (right). A typical result of three performed experiments is shown. (D) *In vitro* Colony formation assay showed the effect of HDAC2 inhibition on the growth of cell colonies after three weeks incubation. Quantification of colony numbers shown is mean ± SD of three independent experiments (top). The representative scan of 0.5% crystal violet-stained cells (bottom). (E) Tumor growth of Hep3B cell xenografts. (F) Representative tumors obtained at sacrifice after 52 days of growth (upper left) and mice (upper right). The tumor volume at sacrifice was presented as bar graph. Values shown are mean ± SD, n = 5 for Mock; n = 3 for HDAC2KD. Data in (B, D, E and F) were analyzed by Student's t-test. * p<0.05, Hep3B_HDAC2KD vs Hep3B_Mock.

Since our results suggest that transient knockdown of HDAC2 exerts potent anti-tumor activity, we next investigated if sustained suppression of HDAC2 can attenuate tumorigenic potential of HCC cells *in vitro* and *in vivo*. For the analyses of experimental tumorigenesis, Hep3B cells that carrying minimal expression of HDAC2 was established by transfection of HDAC2 shRNA (Hep3B_HDAC2KD). The functional inactivation of HDAC2 was validated by the confirmation of selective induction of p16^INK4A^ and p21^WAF1/Cip1^, and suppression of CDK2, cyclin D1 and PCNA in Hep3B_HDAC2KD cells (Figure 6A).

Then, we assessed the growth rate of Hep3B_HDAC2KD cells, and observed that Hep3B_HDAC2KD cells exhibited reduced growth rate compared to Hep3B_Mock or Hep3B mother cell line (Figure 6B, p<0.05). We also investigated cell cycle analysis with the established cell lines. For a more precise analysis of cell cycle progression, the established cell lines were treated with nocodazole to synchronize all the cells in G2/M phase. We then analyzed the proportion of G1 phase cells after release from the nocodazole block. We observed that the proportion of G1 phase cells (2n) in Hep3B_HDAC2KD were increased at all time points after release compared to the control (Hep3B_Mock) (Figure 6C). These results demonstrate that the proliferative defect and/or growth retardation of Hep3B cells by HDAC2 depletion is contributed by specific G1 arrest in cell cycle regulation.

We next performed *in vitro* colony formation assay, and found that the sustained suppression of HDAC2 led to the reduced clonal cell growth (Figure 6D, p<0.05). Finally, to demonstrate that the aberrant regulation of HDAC2 contributes to the oncogenic property of cells *in vivo*, we subcutaneously injected these cell lines into SCID mice. The overall tumor growth was significantly reduced in Hep3B_HDAC2KD (Figure 6E, p<0.05). The average tumor volume at sacrifice was much smaller in the group injected with Hep3B_HDAC2KD than Hep3B_Mock cells (Figure 6F, p<0.05). For tumor incidence, Hep3B_Mock cell line group bore 5 tumors in all 5 injected animals, but Hep3B_HDAC2KD cell line group exhibited 3 tumors in 5 injected animals.

### HDAC2 regulates p21^WAF1/Cip1^ transcriptional activity via Sp1-binding site enriched proximal region of p21^WAF1/Cip1^ promoter

Several studies have shown that HDAC inhibitors strongly activate the expression of p21^WAF1/Cip1^ through enhanced histone acetylation of p21^WAF1/Cip1^ promoter including Sp1-binding site, which releasing the repressor HDAC from its binding [16], [17]. Our present data also indicate that p21^WAF1/Cip1^ is induced by HDAC2 inactivation in Hep3B cells (Figure 3D, 4A and 5B). This implies that histone deacetylase activity is required to suppress p21^WAF1/Cip1^ transcription activity. Therefore, to investigate whether HDAC2 selectively regulates p21^WAF1/Cip1^ expression through the Sp1-binding site, we performed the reporter assay with reporter constructs described in materials and methods.

**Figure 7:**
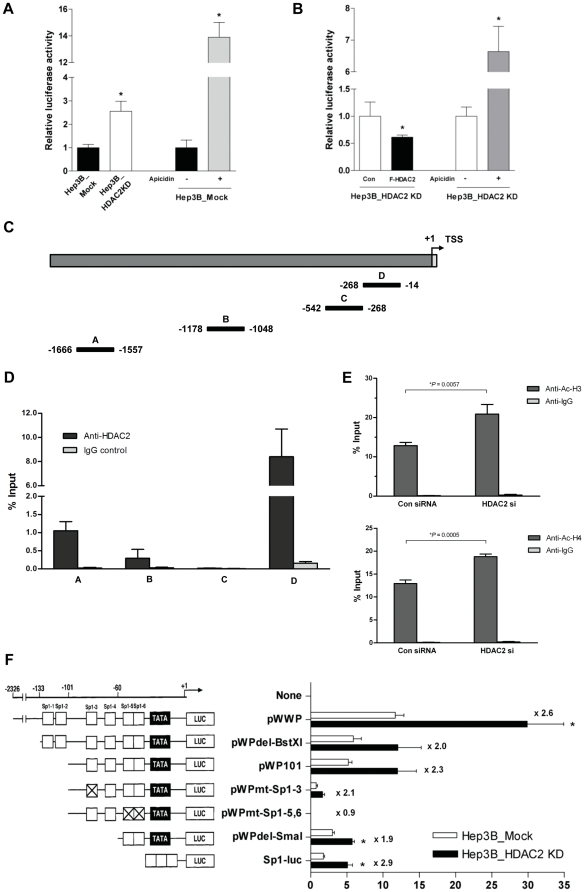
HDAC2 regulates p21^WAF1/Cip1^ transcription via Sp1-binding sites in the p21^WAF1/Cip1^ promoter. (A) HDAC2-deficient (Hep3B_HDAC2KD) or Mock stable cell line (Hep3B_Mock) was transfected with a reporter construct containing the human wild-type p21 promoter (−2326 to +1) linked to a luciferase reporter gene (pWWP-Luc). For the positive control, 1 µM of Apicidin was treated on the mock cell line for 24 h and luciferase activity was measured. The luciferase activity from the mock cells transfected with pGL3 basic vector was arbitrarily defined as 1.0. (B) Ectopic expression of HDAC2 suppressed the p21^WAF1/Cip1^ transcriptional activity. HDAC2-deficient stable cell line (Hep3B_HDAC2KD) was transfected with FLAG-epitope tagged HDAC2 expression vector (F-HDAC2) or control vector (Con). After 24 h incubation, pWWP-Luc vector was also transfected. The promoter activity was measured and normalized as mentioned above. (C) A schematic of the p21^WAF1/Cip1^ promoter depicting the regions analyzed by ChIP-qPCR (black bars, A-D). (D) The association of HDAC2 in the p21^WAF1/Cip1^ promoter was assessed by the amplification of each region immunoprecipitated with HDAC2. The amount of DNA precipitated by either anti-HDAC2 or control IgG was expressed as percentage of the total input genomic DNA. The result of four independent experiments was shown as mean ± SD. (E) Acetylations of histone H3 and H4 associated with the proximal p21^WAF1/Cip1^ promoter was increased by inhibiting association of HDAC2. Hep3B cells were transiently transfected with control (Con) or HDAC2 siRNA for 48 h and subjected to ChIP-qPCR analysis using acetyl-histone H3 (anti-Ac-H3) and H4 (anti-Ac-H4) antibody or control IgG. Precipitated genomic DNA was amplified for the proximal promoter of the *p21* locus (region D represented in Figure 7C) by real-time PCR. The amount of precipitated DNA was expressed as percentage of the total input genomic DNA. The result of three independent experiments was shown as mean ± SD. (F) The indicated constructs, pWWP, pWPdel-BstXI, pWP101, pWPmt-Sp1-3, pWPmt-Sp1-5,6, pWPdel-SmaI and Sp1-luc were transiently transfected into each Hep3B stable cells. The promoter activity was measured, and fold induction by HDAC2 depletion is calculated. On the left, the scheme of each construct was shown. Data in (A, B and F) were analyzed by Student's t-test. * p<0.05.

Firstly, wild-type p21^WAF1/Cip1^ reporter construct was transfected into Hep3B_HDAC2KD cells, and its activity was compared with that of Hep3B_Mock (left two columns in Figure 7A, p<0.05). The Hep3B_Mock cells were also treated with Apicidin, a HDAC inhibitor, as a positive control for induction of p21^WAF1/Cip1^ transcription (right two columns). The Hep3B_HDAC2KD cells exhibited significant induction of luciferase activity compared with Hep3B_Mock. Inversely, ectopic expression of HDAC2 to Hep3B_HDAC2KD cells resulted in the suppression of p21^WAF1/Cip1^ transcriptional activity compared with control (Figure 7B, p<0.05). These results imply that the endogenous expression of p21^WAF1/Cip1^ is selectively regulated by HDAC2 at the transcription level in Hep3B cells. Next, to validate this implication, we performed chromatin immunoprecipitation assay with quantitative PCR (ChIP-qPCR) for indicated p21^WAF1/Cip1^ promoter region (Figure 7C and 7D). From this, we found that HDAC2 is associated with the proximal region of the p21^WAF1/Cip1^ promoter (region D in Figure 7C). This result was further validated by ChIP-qPCR analysis with the same promoter region of p21^WAF1/Cip1^ (region D) that the acetylation status of histone H3 and/or H4 is enhanced by targeting of HDAC2 (Figure 7E). Additionally, as there are enriched Sp1-biding sites in region D, we next investigated whether HDAC2 suppresses p21^WAF1/Cip1^ expression via Sp1-binding sites on the p21^WAF1/Cip1^ promoter. We found that Hep3B_HDAC2KD cells exhibited increased luciferase activity in the presence of Sp1-binding sites, at least containing Sp1-3 to Sp1-6 (pWWP, pWPdel-BstXI and pWP101). However, p21^WAF1/Cip1^ transcription activity was not induced by mutant forms of Sp1-5,6 (pWPmt-Sp1-5,6) in Hep3B_HDAC2KD cells. Interestingly, p21^WAF1/Cip1^ transcription was still induced by two mutant plasmids (pWPdel-SmaI and Sp1-luc) having two or three tandem repeats of Sp1-binding site near the TATA box or transcription start site. Collectively, these results suggest that Sp1-binding site enriched on the proximal region of p21^WAF1/Cip1^ promoter are expected to be an important site regulating p21^WAF1/Cip1^ transcription by HDAC2 in Hep3B cell (Figure 7F, p<0.05).

## Discussion

Accumulating evidences has suggested that HDACs regulate the expression and activity of numerous proteins involved in both cancer initiation and progression [1], [4]. Furthermore, several studies have shown that certain HDAC families are aberrantly expressed in tumors and have redundant function in cancer development [3], [18]. Consistently, targeted-disruption of HDAC2 elicited growth arrest and apoptosis of certain human cancer cells [7], [9], [19], [20], [21], [22]. However, there was no investigation about the role of HDAC2 in liver tumorigenesis until now.

Thus, we assessed HDAC2 expression in a subset of human HCC tissues. From this, we found that HDAC2 is deregulated and overexpressed in all the 10 selected HCCs (Figure 1). Moreover, we confirmed the aberrant expression of HDAC2 in randomly selected another 20 pairs of HCCs (Figure S3). This implies that the deregulation of HDAC2 may be required for initiating and/or developing HCC. With this result, the former report suggesting that the activation of Wnt signaling induced HDAC2 expression and contributed to colon cancer development [7] led us to investigate HDAC2 regulation on enhanced Wnt signaling in HCC. However, our results revealed no association between HDAC2 overexpression and Wnt signaling. This suggests that unlike in colon cancer, transcriptional deregulation of HDAC2 in liver cancer is not affected by Wnt pathway.

Therefore, we then abrogated HDAC2 overexpression to explore tumorigenic functions of HDAC2 in liver tumorigenesis. Our results demonstrated that HDAC2 inactivation caused the suppression of tumor cell growth in various liver cancer cell lines (Figure 2 and S2). Interestingly, although a previous study has demonstrated that HDAC2 inhibition increased apoptosis in HeLa cervical cancer cells [20], our results indicated that HDAC2 inactivation did not induce apoptosis, while it strongly induced G1/S arrest in cell cycle transition.

Many previous reports have suggested that HDAC-mediated repression of genes can cause uncontrolled cell growth as HDACs repress the transcription of cyclin-dependent kinase inhibitors (CDKIs), allowing continued proliferation. Our study demonstrated that HDAC2 depletion selectively induced p16^INK4A^ and p21^WAF1/Cip1^, thereby resulting in the inhibition of G1/S transition of cell cycle (Figure 4A). In addition, HDAC2 inhibition also suppressed the expression of cyclin D1/CDK4 complex and CDK2 in Hep3B cells. Although it is not clear whether p16^INK4A^ and p21^WAF1/Cip1^ suppress the transcriptional activation of these molecules or HDAC2 directly regulates them, it is obvious that this synergistic regulation of CDK/cyclin complex and its specific CDKI in G1/S transition suggests a potent role of HDAC2 in the cell cycle regulation of liver tumorigenesis.

Orderly progression through the cell cycle checkpoints involves coordinated activation of the CDKs in the presence of associated cyclins, which phosphorylates target substrates including members of the “pocket protein” family. One of these, Rb protein (pRb) is phosphorylated sequentially by both the cyclin D1/CDK4 and cyclin E/CDK2 complexes. Our results demonstrated that the cooperative regulation of CDKI and its specific cyclin/CDK complex by HDAC2 caused the hypophosphorylation status of pRb, and as consequently, down-regulated some of E2F/DP1 target genes that may be necessary in G1/S transition (Figure 4B).

HDACs are known to function by interacting with tumor suppressor genes such as p53, pRb and BRCA1 [23], [24], [25]. For example, HDAC1 is necessary for the repression of E2F target genes by pRb [23]. Alternatively, the deacetylation of non-histone proteins such as p53 may also play a role in controlling the cell cycle dynamics. Unlike this suppressive role of HDACs on pRb and its interaction with E2F/DP transcription, our data suggest that HDAC2 act as a potent modulator regulating the expression level of CDK inhibitor, cyclins and CDKs in the G1/S transition. In addition, we noted that both VPA treatment and HDAC2 depletion exerted anti-mitogenic activity through the induction of p21^WAF1/Cip1^, but had different effects on transcriptional regulation of cell cycle components in Hep3B cells (Figure 3D). Indeed, VPA treatment strongly induced p21^WAF1/Cip1^ and suppressed CDK4 expression similar to that with HDAC2 inhibition, but it did not affect the E2F/DP1 target gene expression such as CDC2, PCNA or E2F1 (Figure S4). In addition, from whole gene expression microarray experiment, we were able to identify large-number genes that were differentially expressed by HDAC2 inactivation. The molecular signature that was recapitulated as mitotic gene elements on HDAC2 inactivation included up-regulation of cell cycle inhibitors and down-regulation of cyclins, CDKs and E2F/DP1 target genes (Figure 5A). This result indicates that the targeted-disruption of HDAC2 exerts anti-tumor activity with different spectrums of underlying mechanisms of HDAC inhibitors.

Although there are some reports that HDAC2 expression is dysregulated in cancer cells, only limited numbers of articles have shown *in vivo* analysis of HDAC2 in cancer. In this study, we successfully established stable cell line that sustained limited expression of HDAC2 in Hep3B cells. This cell line (Hep3B_HDAC2KD) exhibited reduced tumor cell growth, retardation of G1/S transition and loss of transforming potentials (Figs. 6A-D). Interestingly, we also found that HDAC2 suppression augmented cellular senescence by which might be the induction of p16^INK4A^ in Hep3B_HDAC2KD cells (Figure S5). With these *in vitro* anti-tumor effects of HDAC2 inhibition, we demonstrated a remarkable suppression of tumor mass growth *in vivo* and a lower tumor incidence by using these cell lines in an experimental mouse xenograft model for cancer (Figs. 6E and 6F).

Dysregulation of both p21^WAF1/Cip1^ expression and HDAC function are commonly observed in a variety of human cancers. It is not clear whether HDAC overexpression leads to the epigenetic suppression of p21^WAF1/Cip1^ *per se* or if other processes also support this phenomenon. However, these mechanisms are closely interconnected, act in a synergistic manner, and offer a growth advantage to the tumor cells. In this respect, our results demonstrated that the suppression of HDAC2 induces p21^WAF1/Cip1^ via Sp1-binding site enriched proximal region of p21^WAF1/Cip1^ promoter (Figure 7). Although it's not clear that HDAC2 suppresses p21^WAF1/Cip1^ through Sp1-binding site by forming a complex with other molecules, it is clear that HDAC2 suppression causes its release from the proximal region near the TATA box (Sp1-5,6) at the p21^WAF1/Cip1^ promoter leading to a loss of repression and an induction of transcription (Figure 7).

In conclusion, we have demonstrated that targeted-disruption of HDAC2 revealed strong anti-proliferative effects on human HCC *in vitro* and *in vivo*. Aberrant actions of HDAC2 disturbed homeostasis via dysregulation of gene expressions of cell cycle components in HCC. Here, we propose that the aberrant regulation of HDAC2 and its epigenetic regulation of gene transcription in cell cycle components play an important role in the development of HCC. This may contribute to tumor cells having mitogenic potential in the initiation and progression of HCC, thereby providing novel targets for therapeutic intervention.

## Materials and Methods

### Ethics Statement

Total thirty hepatocellular carcinoma tissues with their corresponding normal were obtained from Yonsei University, School of Medicine, Seoul, Korea. Informed consent was provided according to the Declaration of Helsinki. Written informed consent was obtained from all subjects, and the study was approved by Ethics Committee of the Catholic University of Korea, College of Medicine (IRB approval number; CUMC09U119). For animal study, all animal experiments were performed in compliance with the guidelines of the Institutional Animal Care and Use Committee (IACUC) of Department of Laboratory Animal, College of Medicine, The Catholic University of Korea (approval number; CUMC-2009-0050-03).

### Plasmid preparation

The following plasmids have previously been described: pME18S-FLAG-HDAC2, which expresses FLAG epitope-tagged HDAC2 [26]; a minimal luciferase reporter plasmid, Sp1-luc [27]; pWWP, which was generated by subcloning the 2.4 kbp human wild-type p21^WAF1/Cip1^ promoter into pGL3-Basic reporter vector, and three deletion-formed plasmids (pWPdel-BstXI, pWP101 and pWPdel-SmaI) and two mutant plasmids which have mutation on specific Sp1 binding site (pWPmt-Sp1-3 and pWPmt-Sp1-5,6) [28]. The pSilencer^TM^3.1-H1 neo plasmid (Ambion, Austin, TX, USA) was used for the construction of shRNA-encoding plasmids. Two pairs of oligonucleotides were annealed and subcloned into the vectors, respectively (Table S1).

### Cell culture and siRNA transfection

Human HCC cell line HepG2 (wt p53), Hep3B (p53 null), PLC/PRF/5 (mt p53) and immortalized liver cell lines (Chang and THLE-3) were obtained from the ATCC (Manassas, VA, USA). The SNU-449 cell line, which harbors p53 mutation at exon 5, was also purchased from the Korean Cell Line Bank (KCLB). siRNA transfection was performed with Oligofectamine or Lipofectamine2000 (Invitrogen, Carlsbad, CA, USA) according to the manufacturer's instructions. Hep3B cells were transfected with validated human β-catenin, c-Myc, cyclin D1 or two pre-designed HDAC2 siRNAs (Table S2) (Ambion) at a final concentration of 100 nM. Scrambled siRNA were also purchased and used as a control.

### Stable expression of HDAC2 shRNA

Cells were transfected with 1 ug of HDAC2-shRNA Expression vector, pSilencer-HDAC2-#1 or pSilencer-HDAC2-#2. At 48 h post-transfection, Geneticin (G418 from Invitrogen) was added to the medium (0.5 mg/mL), and G418-resistant colonies were selected. The medium was changed every 2 days. About 3 weeks later, the G418-resistant cells were isolated as single colonies and separately expanded.

### qRT-PCR

Total RNA was isolated by using TRIzol (Invitrogen) as described by the manufacturer. cDNA was generated by Transcriptor First Strand cDNA Synthesis Kit (Roche Applied Science, Indianapolis, IN, USA). Relative levels of specific mRNA were determined with a SYBR Green chemistry system. All PCRs were performed with the iQ™5 Real-Time PCR Detection System (Bio-Rad Laboratories, Philadelphia, PA, US) according to the manufacturer's instruction. The GAPDH (glyceraldehyde-3-phosphate dehydrogenase) gene was used as a control gene for normalization. PCR primers used were listed in Table S3.

### Western blotting

Cells were lysed using RIPA buffer (25 mM Tris-HCl pH 7.6, 150 mM NaCl, 1% NP-40, 1% sodium deoxycholate, 0.1% SDS, 100 µg/mL PMSF, 1 µg/mL aprotinin and 0.5% sodium orthovanadate). SDS-PAGE and immunoblotting were subsequently performed. Antibodies used were listed in Table S4. The ECL reagent was used and chemiluminescence detected (GE Healthcare).

### Cell growth assay

Trypan blue dye exclusion assay was used to determine the number of viable cells present in a cell suspension. One part of the cell suspension was mixed with one part of 0.4% trypan blue solution, and allowed to incubate at room temperature for 3****min. The trypan blue/cell mixture was then applied to a hemocytometer, and unstained (viable) and stained (non-viable) cells were counted separately using binocular microscope. 3-(4,5-Dimethylthiazol-2-yl)-2,5-diphenyltetrazolium bromide (MTT) assays were also conducted to measure the relative number of viable cells. At indicated time points, medium was replaced with the fresh medium supplemented with MTT (0.5 mg/ml) (Sigma-Aldrich, St. Louis, MO, USA). Absorbance was measured using a multilabel plate reader (VICTOR3^TM^, PerkinElmer, Bridgeport Avenue Shelton, CT, USA) at a wavelength of 570 nm. Experiments were repeated at least three times.

### Cell proliferation assay

Thymidine incorporation was determined using [methyl-^3^H]-thymidine (New England Nuclear Life Science). Briefly, after transfection as described above, [methyl-^3^H]-thymidine (1 µCi/mL) was added to the culture media 10 h prior to each analysis. At 48 and 72 h post-transfection, the cells were washed with PBS and 5% TCA, and lysed using lysis buffer (0.5 M NaOH, 2% SDS). Aliquots were used to determine thymidine incorporation on a scintillation counter LS6500 (Beckman Coulter, Fullerton, CA, USA).

### Detection of apoptotic cells

Quantification of apoptotic cells was performed using Annexin V-FITC Apoptosis Detection Kit according to the manufacturer's specifications (BD Biosciences, San Jose, CA, USA). Cells were analyzed using FACSCalibur flow cytometry and CellQuest^TM^ software (BD Biosciences) to determine the percentage of apoptosis.

### Cell cycle analysis

Cells were trypsinized, washed with cold PBS, and fixed in pre-chilled 70% ethanol at −20°C overnight. For measurement of DNA content, cells were stained with Propidium Iodide (PI) solution (50 µg/mL PI, 100 µg/mL RNase A, 0.05% Triton X-100 in PBS), and incubated at 37°C in the dark for 30 min. DNA content was examined by flow cytometry using FACSCalibur (BD Biosciences) with FlowJo software (Tree Star).

### Microarray analysis

For the large-scale gene expression profiling, HDAC2 gene silencing was performed by transfecting Hep3B cells with HDAC2 shRNA expression plasmids for 48 hours as described above. Total RNA was extracted from three independent sets of experiments using TRIzol Reagent (Invitrogen), followed by clean up on Ambion columns (Illumina Total-Prep RNA Amplification Kit, Ambion). For each experimental condition, an RNA pool was obtained by mixing equal quantities of total RNA from each of the three independent RNA extractions. RNA quality control was performed with Experion^TM^ system (Bio-Rad). Microarray analysis was performed by using HumanHT-12 V4 Expression BeadChip (Illumina, Inc., San Diego, CA). Biotinylated cRNA was prepared by using the MessageAmp kit (Ambion, Inc., Austin, TX). Hybridization and scanning of the chips were performed according to the standard protocol (Illumina). The primary microarray data are available in the GEO database (GSE32070).

### Cell synchronization

Stable cell lines were synchronized in prometaphase by treatment with 100 ng/mL nocodazole for 18 h, and then released from the drug-induced cell cycle arrest. After three washes with PBS, cells were cultured in fresh medium for different times as indicated in the experiment.

### Clonogenic assay

Clonogenic cell growth *in vitro* was assessed according to the previously described method [29]. Briefly, each cell line (Mock or HDAC2 knockdown Hep3B cells) was seeded in 6-well plate (1000 cells/well). After two weeks, colonies are fixed with formaldehyde (1.0% v/v) for 30 min at room temperature, and stained with crystal violet (0.5% w/v). The stained colonies were counted by using the clono-counter software [30].

### Tumor xenograft experiments

For xenograft tumorigenesis assay, 5×10^6^ cells of the indicated cell lines were suspended with 0.2 mL PBS (pH 7.4) and mixed with 20% (v/v) Matrigel. Cell suspensions were subcutaneously inoculated in 5 week-old SCID mice. Tumor size (*V*) was calculated weekly, using the formula, *V* (mm^3^)  = 0.52× (width)^2^× (length).

### ChIP-qPCR analysis

Chromatin preparation was performed by using Chromatin prep module (Pierce, Rockford, IL, USA). In brief, freshly prepared 16% formaldehyde was added to the cells (2×10^6^ cells/ChIP reaction) at a final concentration of 1%. Cells were incubated at room temperature for 10 min, then excess formaldehyde was quenched by addition of glycine (0.5 M). After washing twice, the cells were scraped into 1 ml cold PBS containing 1× Halt Cocktail. The cells were pelleted and then resuspended in 100 µl of Lysis Buffer 1 containing 1× Halt cocktail. Nuclei were isolated and resuspended in MNase Digestion Buffer containing 1 mM DTT. Cross-linked DNA was digested with 0.25 µl of Micrococcal Nuclease (10 U/µl) at 37°C for 15 min yielding fragments from 200 to 1000 bp and centrifuged at 9,000×g to recover the nuclei. Digested chromatin was resuspended in 50 µl of Lysis Buffer 2, and 5 µl of each was reserved as the 10% total input. Chromoatin immunoprecipitation (ChIP) assay was carried out using Pierce Agarose ChIP kit (Pierce) following manufacturer's protocol. For each IP, diluted chromatin was incubated with antibodies specific for HDAC2, Ac-histone H3 and Ac-histone H4 (Cell signaling, Lake Placid, NY, USA) or normal rabbit IgG at 4°C for 2 h or overnight. Each immunoprecipitated complex was column purified using the agarose resin, and eluted in 150 µl of IP Elution Buffer for 30 min at 65°C with shaking. Reverse cross-linked DNA was purified by using DNA Clean-up Column according to manufacturer's instruction. 5 µl of each of the purified DNA was used as template for 60 cycles of PCR amplification using designated primers (Table S3).

### Luciferase assay

Cells (1.5×10^5^ cells/well) were transfected with 500 ng/well of p21^WAF1/Cip1^ promoter reporter plasmid using Lipofectamine PLUS (Invitrogen). At 24 h post-transfection, the medium was changed to a medium with or without 1 µM apicidin. After 24 h of incubation, the activities of luciferase were measured using Dual-Luciferase Reporter Assay System (Promega, Madison, WI, USA).

### Statistical analyses

All experiments were performed three times at least, and samples were analyzed in triplicate. Results were presented as mean (SD). The statistical difference between groups was assessed by applying Student's t-test for unpaired data, using Microsoft Office Excel (Microsoft, Albuquerque, NM, USA).

## Supporting Information

Figure S1 **Expression of HDAC2 in hepatocelluar carcinoma (HCC) defined by gene expression profiling (GEP).** Analysis by gene expression profiling (GEP) data of HDAC2 expression in patients corresponding to low-grade dysplastic nodule (LGDN), high-grade dysplastic nodule (HGDN) and HCC patients (Edmondson grade G1-3). HDAC2 overexpression was observed with significance in high grade tumor (Edmondson grade G3) (p<0.01).
(TIF)

Figure S2 **HDAC2 inactivation causes growth retardation in liver cancer cell lines.** (A) The expression levels of HDAC2 protein in liver cancer cell lines. Total protein extracts were prepared from the indicated cell lines, and were examined by Western blot analysis for the HDAC2. (B) Targeted-disruption of HDAC2 causes growth retardation of HCC cell lines. Cell viability was determined by MTT assay in the indicated cell lines transfected with either control or HDAC2 siRNA. Cell proliferation was determined by measuring the absorbance at A570 using MTT solution at the indicated time after transfection. Data are expressed as mean ± SD (* p<0.05, ** p<0.01). All measurements were performed in triplicate, and each experiment was repeated at least two times.
(TIF)

Figure S3 **HDAC2 expression is aberrantly overexpressed in human HCC.** Human HCC tissue lysates were prepared from non-tumoral liver tissues (N) or tumors (HCC, HBV-positive, Edmondson grade G3) (T) and immunoblotted with HDAC2 antibody. Cell lysates from Hep3B was used as positive control for HDAC2 expression. The GAPDH was used as a loading control.
(TIF)

Figure S4 **Effects of Valproic acid (VPA) on regulatory components of G1/S cell cycle transition in Hep3B cells.** After 48 hours of VPA treatment to the Hep3B cells at indicated concentrations, cells were harvested and subjected for immunoblotting. The GAPDH was used as a loading control, and a typical result of two performed experiments is shown.
(TIF)

Figure S5 **Sustained suppression of HDAC2 increases cellular senescence of Hep3B cells.** Two stable cell lines (Hep3B_Mock and Hep3B_HDAC2KD) were seeded in 60 mm dish (5×10^5^ cells/well). On the next day, cells were fixed and stained for SA (senescence activated)-β-galactosidase expression using the Senescence Detection kit (Biovision) according to the manufacturer's instructions. Cells were incubated with the SA-β-galactosidase staining solution for 16 hours at 37°C. The staining solution was removed and representative images were acquired on a microscope with a CCD camera. Arrows indicate strong positive cells for SA-β-galactosidase staining. Representative images of Hep3B_Mock (left) and Hep3B_HDAC2KD (right) were shown.
(TIF)

Table S1 **Oligonucleotide sequence for HDAC2 shRNA plasmid construction.**
(XLS)

Table S2 **HDAC2 siRNA sequences used in this study.**
(XLS)

Table S3 **Primer information in this study.**
(XLS)

Table S4 **Antibodies used for immunoblotting.**
(XLS)
