# Supplementary figures and images for: Aberrant Regulation of HDAC2 Mediates Proliferation of Hepatocellular Carcinoma Cells by Deregulating Expression of G1/S Cell Cycle Proteins
Source: PLoS One. 2011 Nov 23;6(11):e28103. doi: 10.1371/journal.pone.0028103 (PMC3223227; doi:10.1371/journal.pone.0028103)

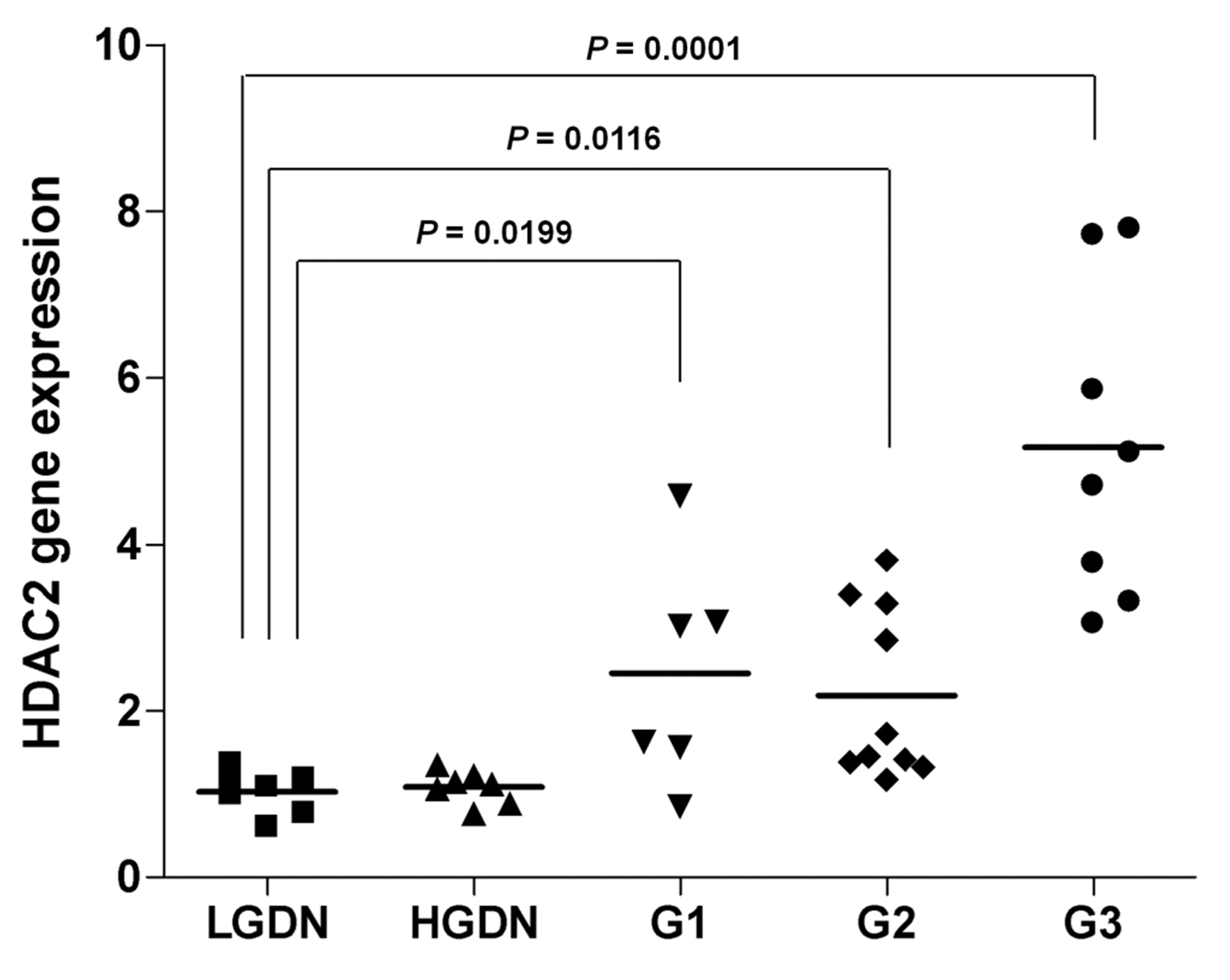

Supplement: Figure S1 — Expression of HDAC2 in hepatocelluar carcinoma (HCC) defined by gene expression profiling (GEP). Analysis by gene expression profiling (GEP) data of HDAC2 expression in patients corresponding to low-grade dysplastic nodule (LGDN), high-grade dysplastic nodule (HGDN) and HCC patients (Edmondson grade G1-3). HDAC2 overexpression was observed with significance in high grade tumor (Edmondson grade G3) (p<0.01). (TIF) [file pone.0028103.s001.tif]

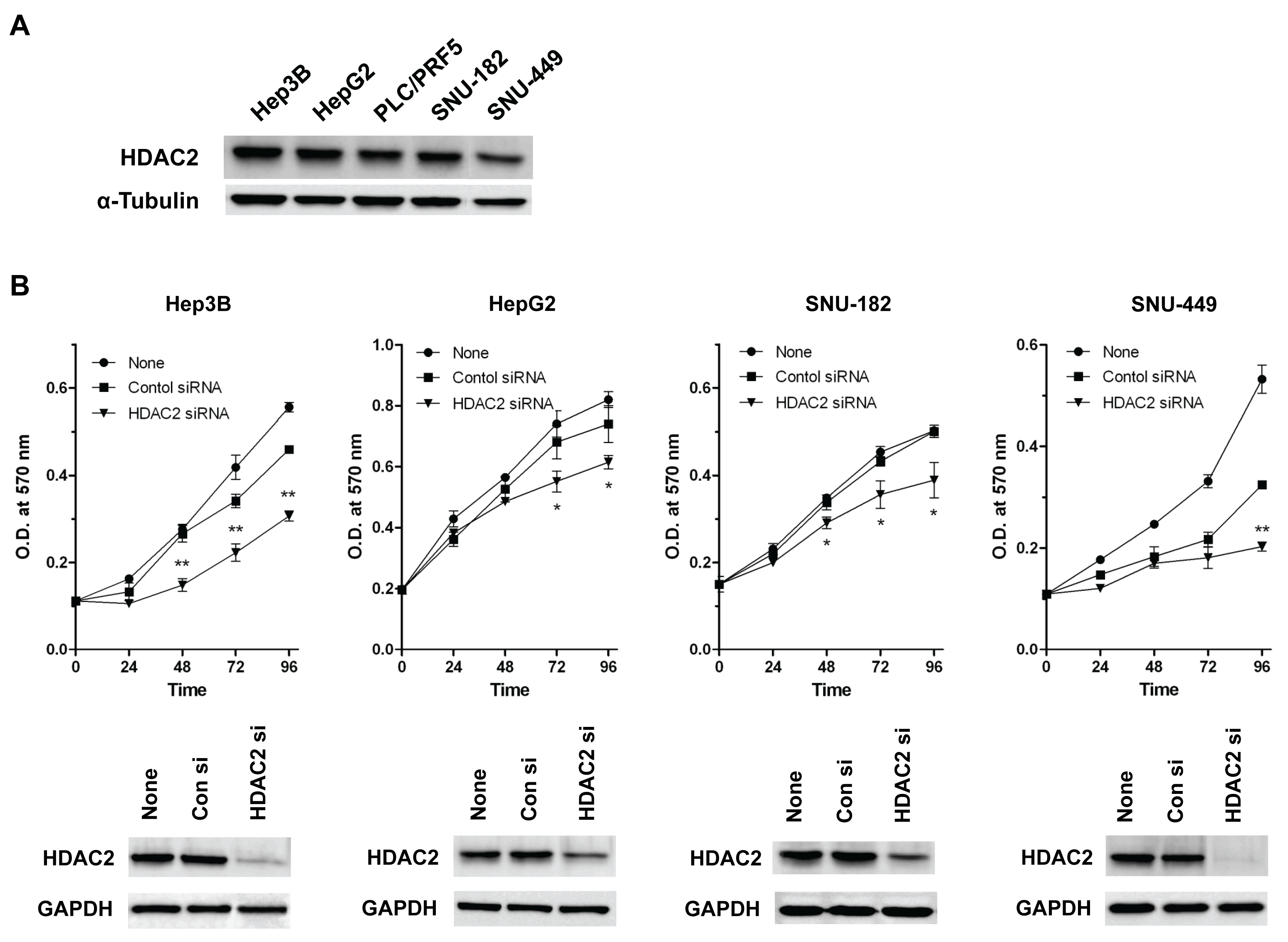

Supplement: Figure S2 — HDAC2 inactivation causes growth retardation in liver cancer cell lines. (A) The expression levels of HDAC2 protein in liver cancer cell lines. Total protein extracts were prepared from the indicated cell lines, and were examined by Western blot analysis for the HDAC2. (B) Targeted-disruption of HDAC2 causes growth retardation of HCC cell lines. Cell viability was determined by MTT assay in the indicated cell lines transfected with either control or HDAC2 siRNA. Cell proliferation was determined by measuring the absorbance at A570 using MTT solution at the indicated time after transfection. Data are expressed as mean ± SD (* p<0.05, ** p<0.01). All measurements were performed in triplicate, and each experiment was repeated at least two times. (TIF) [file pone.0028103.s002.tif]

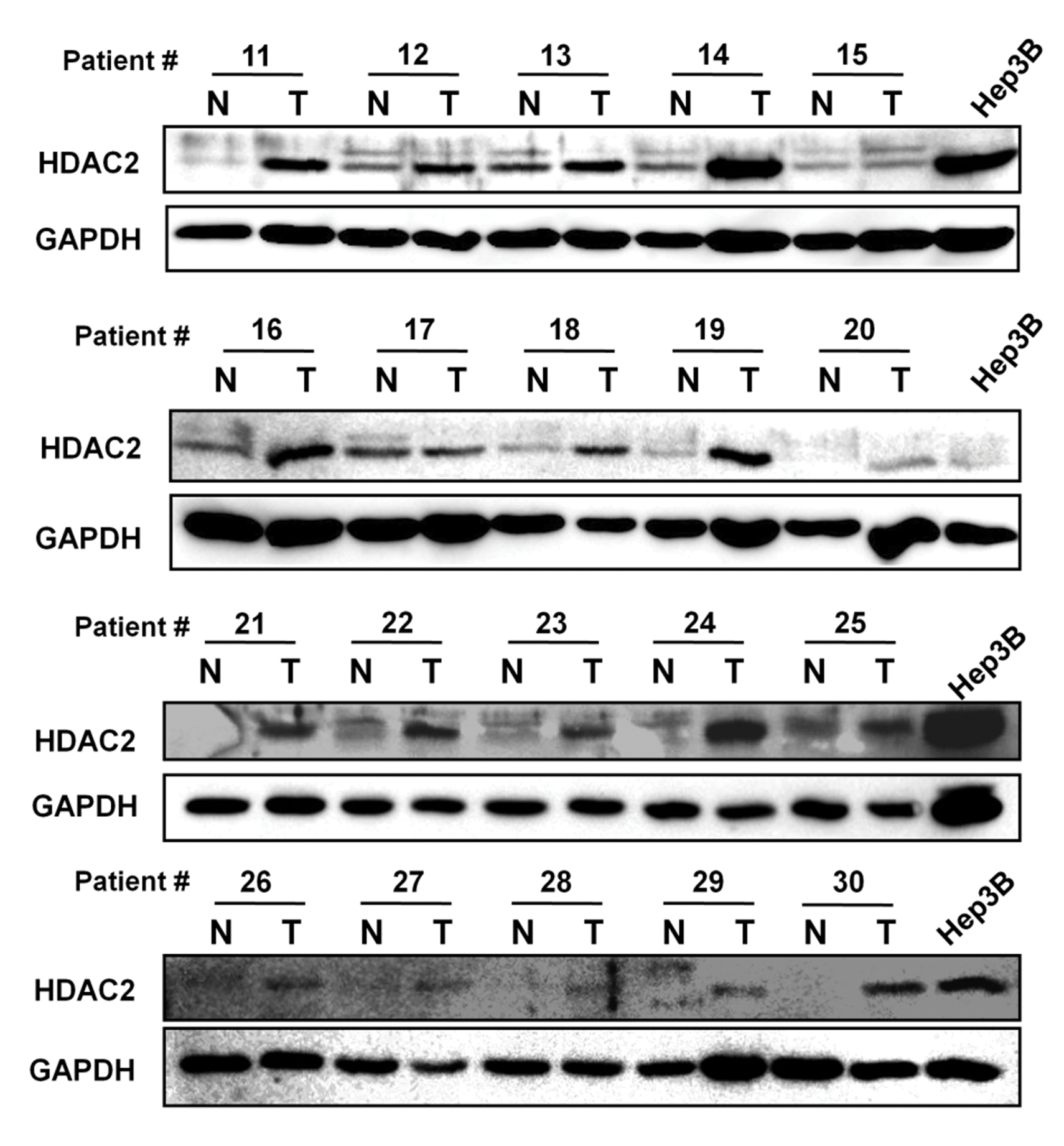

Supplement: Figure S3 — HDAC2 expression is aberrantly overexpressed in human HCC. Human HCC tissue lysates were prepared from non-tumoral liver tissues (N) or tumors (HCC, HBV-positive, Edmondson grade G3) (T) and immunoblotted with HDAC2 antibody. Cell lysates from Hep3B was used as positive control for HDAC2 expression. The GAPDH was used as a loading control. (TIF) [file pone.0028103.s003.tif]

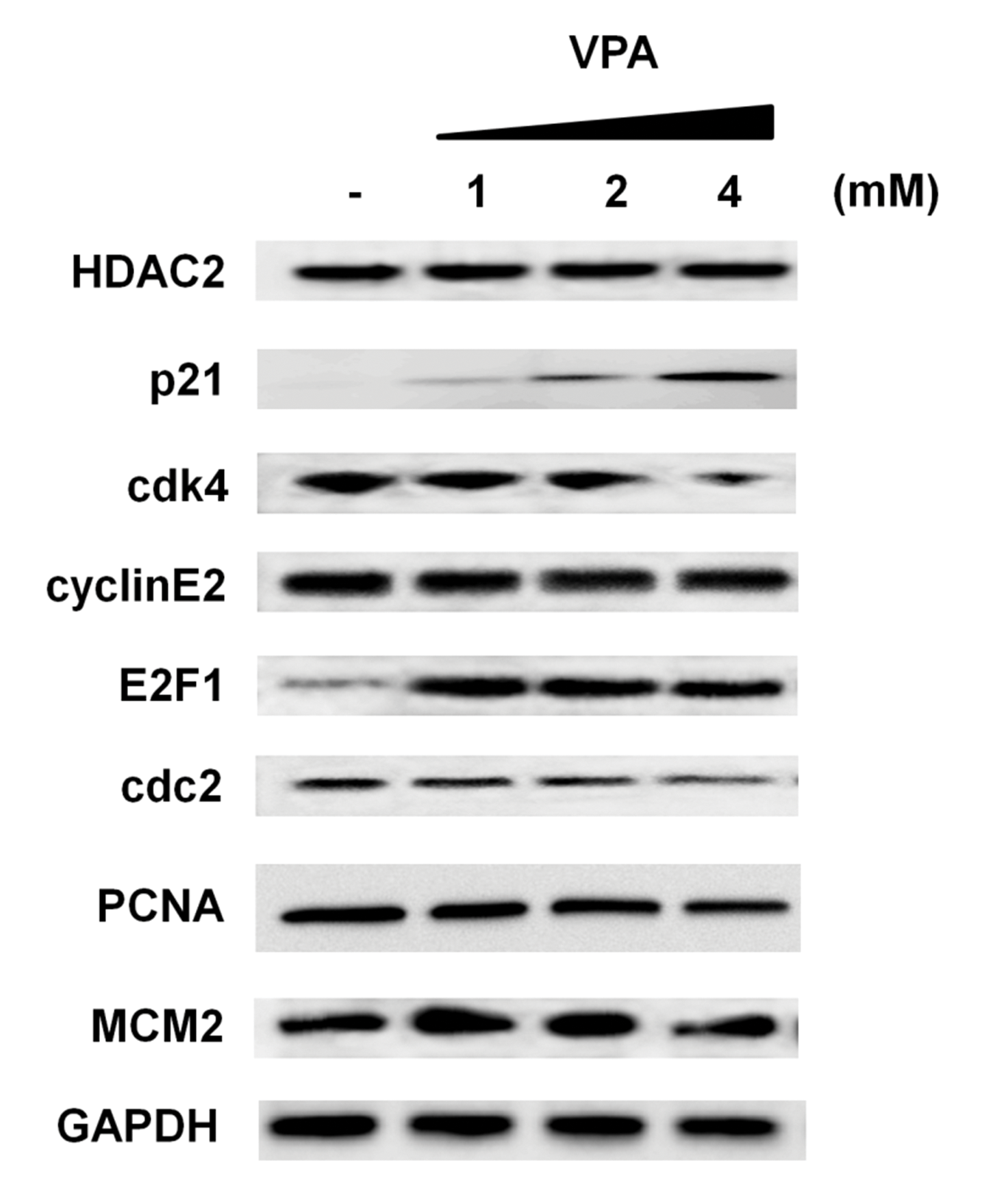

Supplement: Figure S4 — Effects of Valproic acid (VPA) on regulatory components of G1/S cell cycle transition in Hep3B cells. After 48 hours of VPA treatment to the Hep3B cells at indicated concentrations, cells were harvested and subjected for immunoblotting. The GAPDH was used as a loading control, and a typical result of two performed experiments is shown. (TIF) [file pone.0028103.s004.tif]

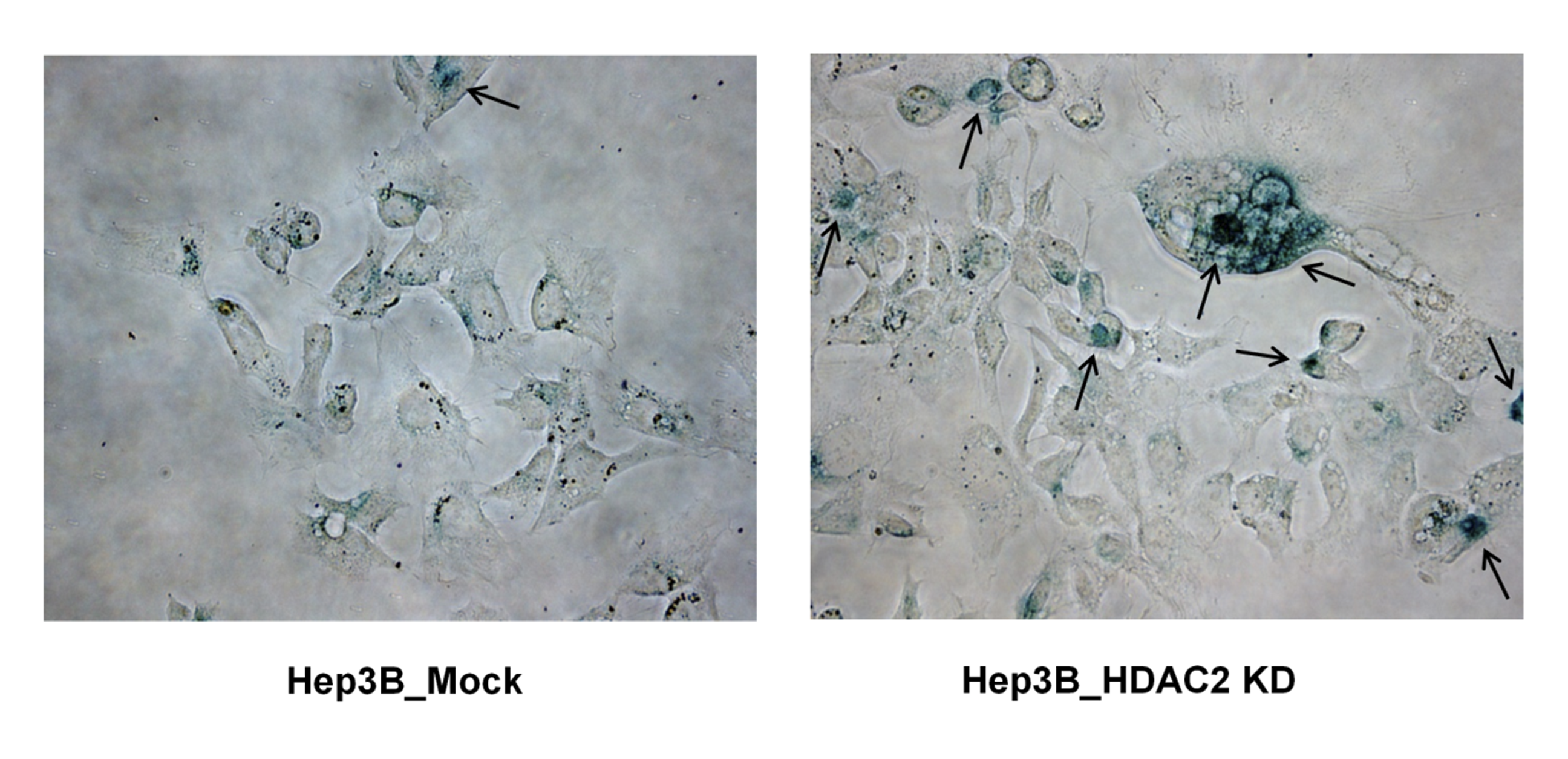

Supplement: Figure S5 — Sustained suppression of HDAC2 increases cellular senescence of Hep3B cells. Two stable cell lines (Hep3B_Mock and Hep3B_HDAC2KD) were seeded in 60 mm dish (5×10^5^ cells/well). On the next day, cells were fixed and stained for SA (senescence activated)-β-galactosidase expression using the Senescence Detection kit (Biovision) according to the manufacturer's instructions. Cells were incubated with the SA-β-galactosidase staining solution for 16 hours at 37°C. The staining solution was removed and representative images were acquired on a microscope with a CCD camera. Arrows indicate strong positive cells for SA-β-galactosidase staining. Representative images of Hep3B_Mock (left) and Hep3B_HDAC2KD (right) were shown. (TIF) [file pone.0028103.s005.tif]
